# Death‐associated protein kinase 3 regulates the myogenic reactivity of cerebral arteries

**DOI:** 10.1113/EP090631

**Published:** 2023-04-21

**Authors:** Sara R. Turner, Abdulhameed Al‐Ghabkari, David A. Carlson, Mona Chappellaz, Cindy Sutherland, Timothy A. J. Haystead, William C. Cole, Justin A. MacDonald

**Affiliations:** ^1^ Department of Biochemistry & Molecular Biology, Cumming School of Medicine University of Calgary Calgary AB Canada; ^2^ Department of Pharmacology & Cancer Biology Duke University School of Medicine Durham NC USA; ^3^ Department of Physiology and Pharmacology, Cumming School of Medicine University of Calgary Calgary AB Canada

**Keywords:** cerebrovascular, myogenic reactivity, vascular smooth muscle

## Abstract

The vascular smooth muscle (VSM) of resistance blood vessels is a target of intrinsic autoregulatory responses to increased intraluminal pressure, the myogenic response. In the brain, the myogenic reactivity of cerebral arteries is critical to homeostatic blood flow regulation. Here we provide the first evidence to link the death‐associated protein kinase 3 (DAPK3) to the myogenic response of rat and human cerebral arteries. DAPK3 is a Ser/Thr kinase involved in Ca^2+^‐sensitization mechanisms of smooth muscle contraction. *Ex vivo* administration of a specific DAPK3 inhibitor (i.e., HS38) could attenuate vessel constrictions invoked by serotonin as well as intraluminal pressure elevation. The HS38‐dependent dilatation was not associated with any change in myosin light chain (LC20) phosphorylation. The results suggest that DAPK3 does not regulate Ca^2+^ sensitization pathways during the myogenic response of cerebral vessels but rather operates to control the actin cytoskeleton. A slow return of myogenic tone was observed during the sustained *ex vivo* exposure of cerebral arteries to HS38. Recovery of tone was associated with greater LC20 phosphorylation that suggests intrinsic signalling compensation in response to attenuation of DAPK3 activity. Additional experiments with VSM cells revealed HS38‐ and siDAPK‐dependent effects on the actin cytoskeleton and focal adhesion kinase phosphorylation status. The translational importance of DAPK3 to the human cerebral vasculature was noted, with robust expression of the protein kinase and significant HS38‐dependent attenuation of myogenic reactivity found for human pial vessels.

## Abstract (highlights)

- **What is the central question of this study?**
  DAPK3 contributes to the Ca^2+^‐sensitization of vascular smooth muscle contraction: does this protein kinase participate in the myogenic response of cerebral arteries?
- **What is the main finding and its importance?**
  Small molecule inhibitors of DAPK3 effectively block the myogenic responses of cerebral arteries. HS38‐dependent changes to vessel constriction occur independent of LC20 phosphorylation, and therefore DAPK3 appears to operate via the actin cytoskeleton. A role for DAPK3 in the myogenic response was not previously reported, and the results support a potential new therapeutic target in the cerebrovascular system.

## 1 INTRODUCTION

The primary function of the arterioles is to regulate flow into downstream capillaries, and they achieve this through contractile tone development (Pugsley & Tabrizchi, 2000). A key component of tone development in the resistance vasculature is the myogenic response, referring to constrictions originating within the vascular smooth muscle (VSM) as a response to stretch. The vascular myogenic response is an inherent property of the resistance arteries and has been well studied in isolated pressurized vessels over the past 25 years (K. S. Hong et al., 2020). This vascular contractile response to increased luminal pressure involves membrane depolarization and Ca^2+^ influx, as well as several Ca^2+^ sensitization mechanisms (as reviewed in: Cole & Welsh, 2011; Davis & Hill, 1999; El‐Yazbi & Abd‐Elrahman, 2017; Hill et al., 2001; K. S. Hong et al., 2020; Jackson, 2021; Schubert et al., 2008).

Despite their relatively large size, the middle cerebral arteries (MCA) and posterior cerebral arteries (PCA) display strong myogenic responses and are the primary regulators of blood flow to the cerebrum (Izzard & Heagerty, 2014). These vessels provide critical homeostatic mechanisms for the maintenance of constant blood flow to the brain, which can easily suffer damage from brief periods of either sub‐ or supra‐optimal perfusion. The myogenic response also maintains capillary hydrostatic pressure despite fluctuations in systemic pressure and shields delicate vessels from damage caused by sudden increases in flow (Shekhar et al., 2017).

The death‐associated protein kinase 3 (DAPK3, also known as zipper‐interacting protein kinase or ZIPK) is a recent addition to a cadre of Ca^2+^/calmodulin‐independent protein kinases involved in Ca^2+^ sensitization mechanisms of VSM (Haystead, 2005; Ihara & MacDonald, 2007). DAPK3 can phosphorylate the 20‐kDa regulatory myosin light chain (LC20) (Borman et al., 2002; Komatsu & Ikebe, 2014; Moffat et al., 2011; Niiro & Ikebe, 2001), as well as the myosin phosphatase targeting subunit (MYPT1) (Endo et al., 2004; MacDonald, Borman et al., 2001) and the 17‐kDa C‐kinase‐potentiated inhibitor (CPI‐17) of myosin phosphatase (MacDonald, Eto et al., 2001; Xu et al., 2010). While strong evidence supports the ability of DAPK3 to regulate the contractile force of isolated VSM (Borman et al., 2002; Carlson et al., 2013, 2018; Endo et al., 2004; MacDonald et al., 2016; MacDonald, Borman et al., 2001; Niiro & Ikebe, 2001; Usui et al., 2012), to date, there have been no examinations of DAPK3 in the vascular myogenic response. Thus, although DAPK3 can regulate VSM contractile force development under various experimental conditions, whether this signalling molecule participates in the physiological myogenic responses of cerebral arteries to increasing intraluminal pressure remains to be investigated.

## 2 METHODS

### 2.1 Ethical approvals

Animals were housed and handled according to the standards of the Canadian Council on Animal Care and protocols (nos AC17‐0064 and AC14‐0018) reviewed by the Animal Care Committee of the Cumming School of Medicine, University of Calgary. Human brain tissue samples were obtained from surgical patients following written informed consent, and in accordance with the guidelines of the *Declaration of Helsinki*. Ethical approval was provided by the University of Calgary and Calgary Health Region Conjoint Health Research Ethics Board (Ethics CHREB ID: 024735).

### 2.2 Reagents

5‐Hydroxytryptamine (5‐HT), acetylcholine (ACh) and phenylephrine (PE) were purchased from Millipore‐Sigma (Oakville, ON, Canada). Anti‐DAPK3 (rabbit polyclonal, ab115695), anti‐focal adhesion kinase (FAK; rabbit monoclonal (EP695Y), ab40794) anti‐pY397‐FAK (rabbit polyclonal, ab39967), anti‐SM22α (rabbit polyclonal, ab14106) and anti‐αSM‐actin (rabbit polyclonal, ab5694) were obtained from Abcam (Cambridge, UK). Anti‐LC20 (mouse monoclonal, sc‐2839) was purchased from Santa Cruz Biotechnology (Dallas, TX, USA). Human DAPK3‐siRNA (am‐4390824) and scrambled siRNA (am‐AM4613) were purchased from Ambion/Thermo Fisher Scientific (Mississauga, ON, Canada). The HS38 and HS94 compounds were synthesized as described previously (Carlson et al., 2013, 2018). Phos‐Tag‐acrylamide was purchased from NARD Chemicals, Inc. (Kobe City, Japan). All other chemicals were reagent grade and were obtained from Millipore‐Sigma or VWR (Mississauga, ON, Canada).

### 2.3 Animals

Male Sprague–Dawley rats (∼300 g) were purchased from Charles River Laboratories (Montreal, QC, Canada) and were housed in the Animal Resource Center at the University of Calgary. Animals were killed by isoflurane inhalation to the point of being fully anaesthetized, followed by decapitation by guillotine. Immediately following killing, organs and tissues required for study were removed by gross dissection and placed into room temperature normal Krebs's buffer (NKB) containing 120 mM NaCl, 25 mM NaHCO_3_, 4.8 mM KCl, 1.2 mM NaH_2_PO_4_, 1.2 mM MgSO_4_, 11 mM glucose and 1.8 mM CaCl_2_ (pH 7.4).

### 2.4 Vessel isolation and mounting for pressure myography

PCA vessels were collected from their origin within the circle of Willis to their first major branch point, cleaned of surrounding tissue, and cut into 3–5 mm segments. For myography, vessels were mounted onto one glass cannula in an arteriograph chamber (Living Systems CH‐1‐QT; St Albans, VT, USA) and tied in place with two pieces of silk suture. To denude the vessel of endothelial cells, each vessel was passed fully onto the glass cannula, and then a stream of air bubbles was passed through the lumen of the vessel. After denuding, the other end of the vessel was mounted on the opposite cannula and tied in place with silk suture. The vessel chamber was then connected to the pressure myograph for measurement of vessel diameters (outer and inner diameters: OD and ID, respectively) via an automated edge detection system (IonOptix; Westwood, MA, USA). Vessels were briefly pressurized to 80 mmHg (∼ 1 min) to check for leaks in the system; leaky vessels were discarded. Pressure was then set to 10 mmHg and vessels were warmed to 37°C by a perfusing bath of warm, aerated (with 95% air–5% CO_2_) NKB for a 20 min equilibration period.

### 2.5 Pressure‐induced constriction protocol

Mounted PCA vessels were first tested for the presence of myogenic reactivity since the myogenic response may be lost due to mishandling during dissection and mounting. Vessels were pressurized to 80 mmHg, and the pressure was dropped back to 10 mmHg following development of myogenic constrictions and stabilization of vessel diameters. Confirmation of endothelial removal was made via loss of vasodilatory response to acetylcholine (ACh, 1 μM) in vessels displaying myogenic constriction. The single step to 80 mmHg was repeated up to two additional times to confirm the stability of the myogenic response. The vessel was then subjected to pressure steps in NKB from 10 to 120 mmHg; individual pressure steps lasted for approximately 8 min and allowed for development of stable diameters. Pressure was then returned to 10 mmHg. In cases where DAPK3 inhibitors were tested for effects on the myogenic response, compounds were added to the bath superfusate; the vessel was then incubated at 10 mmHg for 30 min prior to repeating the 10–120 mmHg pressure steps. Pressure was then returned to 10 mmHg and the NKB, with or without inhibitor, was removed and replaced with Ca^2+^‐free Krebs (0 Ca^2+^; possessing the same chemical constituents as NKB, but with no CaCl_2_ and the addition of 2 mM EGTA). Data represent the average vessel diameters during stable development at each pressure step and were expressed as a percentage of the maximal passive vessel diameter in 0 Ca^2+^ solution to account for variations in the sizes of vessels among animals. Some vessels were flash‐frozen with a dry ice–methanol bath; these were transferred to 1.5 ml microtubes, lyophilized for 16 h, and then stored at −80°C.

### 2.6 Human cerebral vessel pressure myography

Brain tissues were collected by the surgeon, placed in cold NKB and then rapidly transferred to the laboratory post‐operatively. Small superficial cerebral pial arteries, approximately 150–250 μm in diameter, were carefully dissected from surrounding tissue and cut into segments to be mounted for pressure myography. Larger superficial cerebral arteries, approximately 300–500 μm in diameter and 1 cm in length, were removed and flash frozen for biochemical assessments.

### 2.7 VSM cell cultures

Coronary artery smooth muscle cells (CASMCs, CC‐2583; Lonza; Allendale, NJ, USA) were maintained and cultured in smooth muscle basal medium (SmGM‐2 BulletKit; CC‐3182; Lonza). The medium was supplemented with hEGF, insulin, hFGF‐B and fetal bovine sserum (FBS). Cells were confirmed to be negative for *Mycoplama* strain contamination and then seeded at density of 3500 viable cells/cm^2^ according to the manufacturer's protocol. Cell viability, morphology and proliferative capacity were routinely examined after recovery from cryopreservation. Passages from 8 to 12 were used in different experimental conditions. In some cases, scrambled‐ or DAPK3‐siRNAs were diluted with siRNA transfection medium (sc‐36868, Santa Cruz Biotechnology) to give a final concentration of 20 nM. The siRNA solutions were gently overlaid onto the cells and incubated for 6–12 h at 37°C. Normal growth medium (2 ml containing two times the normal serum and antibiotic concentrations) was added, and the cells were maintained for 24–48 h until harvest for analyses. Whole cell extracts were prepared from CASMC cultures by first washing cells with phosphate‐buffered saline (PBS; 136.9 mM NaCl, 2.7 mM KCl, 10.1 mM Na_2_HPO_4_ and 1.76 mM KH_2_PO_4_) and then completing cellular lysis with addition of 2× sample buffer (2% (w/v) SDS, 100 mM dithiothreitol, 10% (v/v) glycerol and 60 mM Tris–HCl, pH 6.8) with gentle rocking followed by heating at 95°C.

### 2.8 Western immunoblotting

CASMC whole cell extracts were separated on 12% SDS‐PAGE gels using standard conditions and then transferred to polyvinylidene difluoride (PVDF) membranes (0.45 μm pore size) at 110 V (constant voltage) for 1 h at 4°C using standard Tris–glycine buffers (25 mM Tris, 192 mM glycine, pH 8.3, 20% (v/v) methanol and 0.1% (w/v) SDS).

### 2.9 Immunocytochemistry

CASMCs (2 × 10^5^ cells) were cultured in SmGM‐2 medium with 10% (v/v) FBS at 37°C with 5% CO_2_. Cells were fixed (15 min) in 4% (v/v) paraformaldehyde in PBS and then permeabilized with 0.5% (v/v) Tween‐20 for 10 min. For F‐actin visualization, Alexa Fluor 488–phalloidin was diluted 1:40 in 1% (w/v) BSA in PBS and then incubated with the cells for 1 h at room temperature. Cells were rinsed with PBS, counterstained with 4′,6‐diamidino‐2‐phenylindole (DAPI) for 5 min to detect nuclei, and then examined with an InCell 6000 Imaging System (GE Healthcare, Mississauga, ON, Canada). For visualization, the Imaging System was programmed to complete whole‐well scanning of ten random visual fields from four independent plates of cells. After blocking with 5% non‐fat milk protein, membranes were incubated with primary antibody (1:1000 dilution) for 16 h at 4°C, washed extensively and then incubated with horseradish peroxidase (HRP)‐conjugated anti‐IgG secondary antibody (1:2500 dilution) for 1 h at room temperature. A final washing step was completed and then membranes were developed with enhanced chemiluminescence (ECL) reagent and imaged using an LAS4000 Gel Imager with ImageQuant densitometry software (GE Healthcare).

### 2.10 Western immunoblotting of human vessels

SDS‐PAGE Sample Buffer (50 μl) containing 60 mM Tris–HCl (pH 6.8), 4% (w/v) SDS, 10 mM dithiothreitol, 10% (v/v) glycerol and 0.01% (w/v) bromophenol blue was added to a microtube containing a single vessel. The tubes were centrifuged (1 min; 13,500 *g*) to ensure tissues were fully submerged in Sample Buffer and were then vortexed for 16 h at 4°C. The samples were centrifuged again briefly (1 min; 13,500 *g*), heated to 95°C for 10 min and then stored at –20°C until use. Three‐step western immunoblotting procedures were conducted as previously described (Johnson et al., 2009). Proteins were separated on 12% SDS‐PAGE gels using standard conditions and then transferred to nitrocellulose membranes (0.2 μm pore size; Bio‐Rad Laboratories, Hercules, CA, USA) at 25 V (constant voltage) for 16 h at 4°C using standard Tris–glycine buffers (25 mM Tris, 192 mM glycine, pH 8.3, 20% (v/v) methanol and 0.1% (w/v) SDS). After blocking with 5% non‐fat milk protein, membranes were sequentially incubated with primary antibody (a 1:1000 dilution) for 16 h at 4°C and then biotin‐conjugated goat anti‐rabbit IgG (Millipore‐Sigma/Chemicon, Oakville, ON, Canada) secondary antibody (1:10,000 dilution) for 1 h at room temperature. After extensive washing, membranes were incubated for 30 min at room temperature in HRP‐conjugated streptavidin (Thermo Fisher Scientific (Pierce), Waltham, MA, USA) at 1:20,000 dilution. A final washing step was completed and then membranes were developed with ECL reagent and imaged using an LAS4000 Gel Imager.

### 2.11 LC20 phosphorylation

Phos‐tag SDS‐PAGE was used to monitor LC20 phosphorylation status as previously described (Chappellaz et al., 2018; MacDonald et al., 2016). PCA tissue extracts were resolved with Phos‐tag SDS‐PAGE gels containing 12.5% (w/v) acrylamide, 0.42% (w/v) *N*,*N*′‐methylene bisacrylamide solution with 0.1% (w/v) SDS, 60 μM MnCl_2_ and 30 μM Phos‐tag reagent. Gels were developed at 30 mA/gel in standard Tris–glycine buffer for 70 min. Proteins were transferred to 0.2 μm PVDF membranes and fixed with 0.5% (v/v) glutaraldehyde solution. Membranes were probed with primary anti‐LC20 antibody (1:1000 dilution), incubated with HRP‐conjugated secondary antibody (1:10,000 dilution), developed with ECL reagent and then visualized with an LAS4000 Gel Imager. LC20 phosphorylation stoichiometry was calculated as previously described (MacDonald et al., 2016).

### 2.12 Data analysis

Values are presented as the mean with standard deviation (SD), with *n* indicating the number of vessels from different animals or separate cell culture passages. Data were analysed using GraphPad‐Prism software (GraphPad Software, San Diego, CA, USA). Multiple group comparisons were made as appropriate by two‐way analysis of variance (ANOVA) with Šidák's *post hoc* test or one‐way ANOVA and Tukey's multiple comparison test. Pairwise comparisons were made with Student's *t*‐test (two‐way). Differences were considered statistically significant when *P* < 0.05.

## 3 RESULTS

**FIGURE 1:**
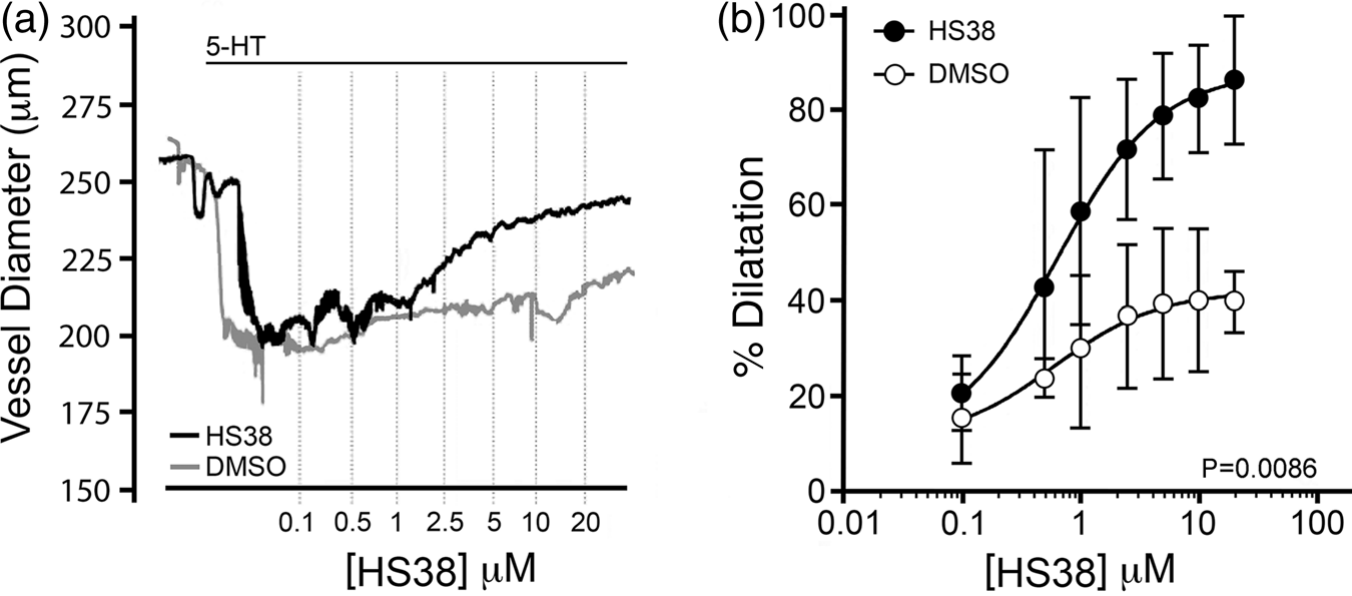
HS38 treatment relaxes rat posterior cerebral arteries constricted with serotonin. Posterior cerebral arteries were mounted at 10 mmHg and constricted with continuous exposure to serotonin hydrochloride (5‐HT, 1 μM). Increasing concentrations of HS38 (0.1–20 μM) were applied to the bath chamber. (a) Representative recordings of the change in outer diameter for 5‐HT constricted vessels subjected to sequential application of HS38. (b) Cumulative data showing vessel constriction in the presence or absence of HS38 relative to the maximum passive diameter observed in 0 Ca^2+^ solution. Vehicle and HS38 treatment groups were found to be significantly different (two‐way ANOVA, *P* = 0.0086). Data are displayed as mean with SD; vessels were obtained from *n* = 4 different male animals.

The PCA was selected as the vessel of choice to evaluate the contribution of DAPK3 to the vascular myogenic response. These arteries, along with the MCAs, display a high degree of myogenic reactivity (Abd‐Elrahman et al., 2017; Colinas et al., 2015). A concentration–response experiment was conducted to confirm the concentration of HS38 required to inhibit DAPK3 in isolated pressurized vessels *ex vivo*. As shown in Figure 1a, vessels were pressurized to 10 mmHg and pre‐constricted with 1 μM 5‐hydroxytryptamine (5‐HT). After a stable vessel diameter was established, HS38 was added to the bath at increasing concentrations (0.1 to 20 μM; dimethyl sulfoxide (DMSO) was used as a vehicle control). Vessels constricted by 17.5 (12.4)% in response to 5‐HT (relative to their initial diameter), with an average diameter change of 52.1 (17.6) μm (Figure 1b). In the vehicle controls, the vessels were also observed to dilate by 20.8 μm or 41.4 (8.9)% by the conclusion of the experiment. However, HS38 was found to significantly dilate the 5‐HT‐constricted vessel beyond that observed for the DMSO control (*P* = 0.0086). In this case, the maximal dilatation with HS38 treatment was 45.6 μm or 87.6 (17.8)% with a calculated EC_50_ of 0.65 μM.

**FIGURE 2:**
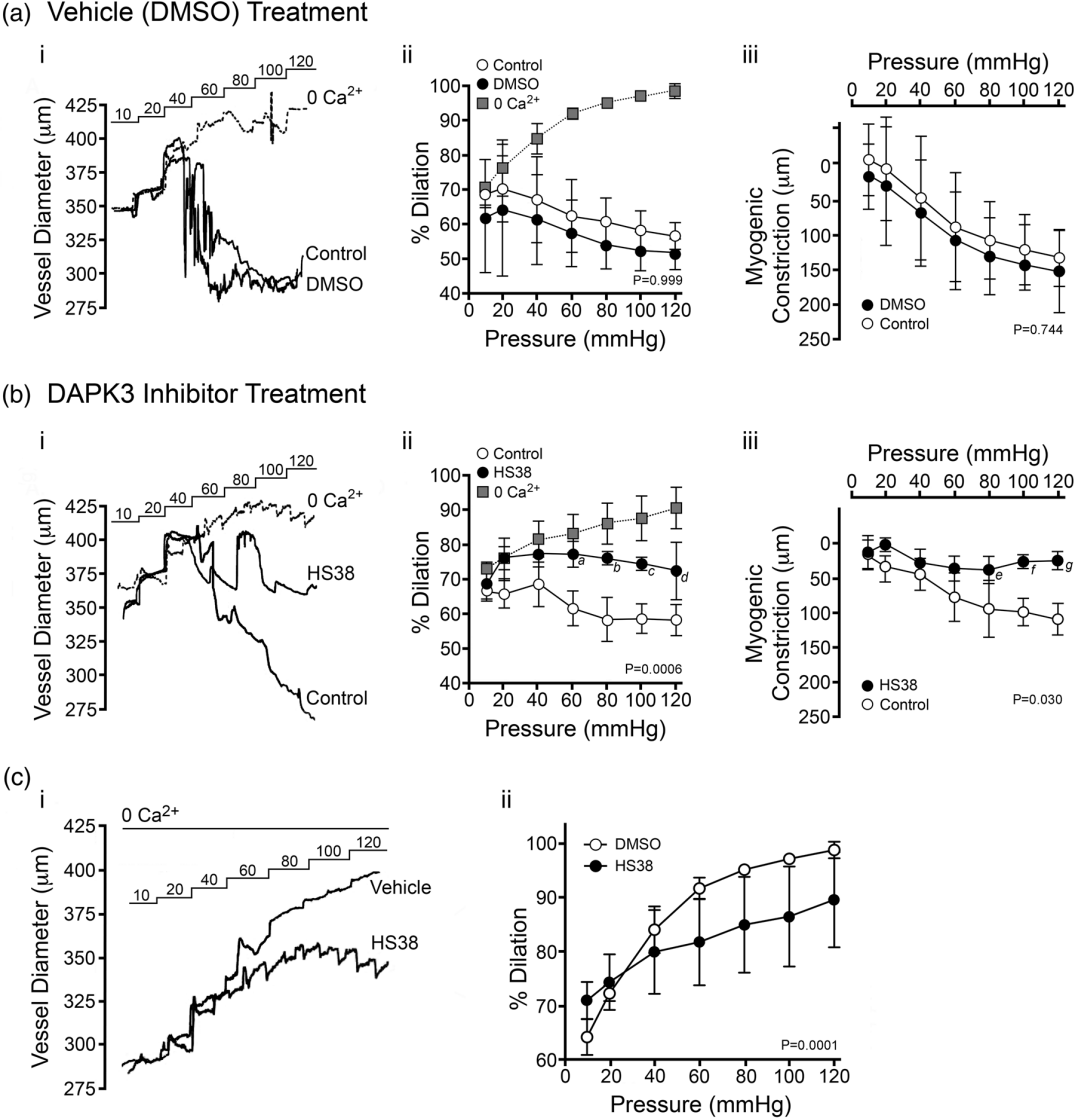
HS38 administration attenuates the myogenic response of rat posterior cerebral arteries and elicits a decrease in passive vessel diameter. The myogenic reactivity of posterior cerebral arteries was monitored in the absence (a, DMSO vehicle) or presence of DAPK3 inhibitor (b, HS38, 10 μM). (i) Representative recordings of the change in outer diameter for vessels subjected to sequential pressure steps (10–120 mmHg) in the presence of normal Krebs buffer (NB), vehicle or DAPK3 inhibitor, and then calcium‐free buffer (0 Ca^2+^). (ii) Cumulative data showing the vessel constriction relative to the maximum passive diameter observed in 0 Ca^2+^ solution. (iii) Magnitude of active myogenic constriction. No significant impact on percentage dilatation (a, ii) or myogenic constriction (a, iii) was identified for vehicle/DMSO treatment; *P* = 0.999 and *P* = 0.744, respectively, by two‐way ANOVA with Šidák's multiple comparisons test. HS38 treatment did have a significant impact on percentage dilatation above 60 mmHg (b, ii) and myogenic constriction above 80 mmHg (b, iii); *P* = 0.0006 and *P* = 0.030, respectively by two‐way ANOVA with Šidák's multiple comparisons tests (*a*, *P* = 0.023; *b*, *P* = 0.009; *c*, *P* = 0.021; *d*, *P* = 0.041; *e*, *P* = 0.023; *f*, *P* = 0.0025; *g*, *P* = 0.0006). Data are displayed as means with SD; vessels were obtained from *n* = 3 different male animals. (c, i) Representative recordings illustrating the change in outer diameter for vessels in calcium‐free Krebs buffer (0 Ca^2+^). Arteries were incubated at 10 mmHg for 30 min in the presence of vehicle (DMSO) or HS38 (10 μM), and then pressure steps (20–120 mmHg) were developed over an additional 60 min. (c, ii) Cumulative data show the vessel constriction relative to the maximum passive diameter observed in 0 Ca^2+^ solution. Vehicle and HS38 treatment groups were found to be significantly different (two‐way ANOVA, *P* = 0.0001). Data are presented as means with SD; vessels were obtained from *n* = 4 different male animals.

Knowing that the application of HS38 was able to attenuate the constriction of PCA vessels by 5‐HT, we examined the effect of this compound on intraluminal pressure‐dependent myogenic reactivity. Vessels were isolated and subjected to three consecutive series of pressure steps (10–120 mmHg): first in normal Krebs buffer, then in Krebs solution containing 10 μM HS38 or DMSO vehicle control, and finally in zero external calcium (0 Ca^2+^) Krebs buffer. In control vessels, myogenic constrictions were initiated at 20–40 mmHg (Figure 2a, i), with maximal active constrictions at 120 mmHg of 148 μm or approximately 60% of the maximal vessel diameter under 0 Ca^2+^ conditions. These results agree with previously published myography studies of the PCA (Kim et al., 2013) and the MCA vessel (Johnson et al., 2009). DMSO had no effect on the myogenic response; neither the percentage dilatation (*P* = 0.999) nor the active constriction (*P* = 0.744) was significantly impacted (Figure 2a, ii and iii, respectively). HS38 treatment markedly reduced the magnitude of myogenic constriction (Figure 2b, i) over a broad range of intraluminal pressures (*P* = 0.0006). The HS38‐treated vessels retained 75.4 (7.4)% of the maximal diameter measured under 0 Ca^2+^ conditions (Figure 2b, ii), and the average change in active constriction was 72.7 μm at 100 mmHg (Figure 2b, iii) when control and HS38‐treated vessels were compared (*P* = 0.030).

It appeared that PCA vessels treated with HS38 did not dilate fully and continued to exhibit some constriction during the final round of pressure steps in 0 Ca^2+^ Krebs buffer, relative to control vessels. Subsequently, rather than standardize data to the maximum passive diameter in 0 Ca^2+^ Krebs buffer at the end of the experiment as is in the literature (Schjorring et al., 2015; Wenceslau et al., 2021), a brief pressure step to 120 mmHg was performed immediately after mounting the PCA, and maximum vessel diameter was recorded prior to the development of any myogenic constrictions. To further interrogate the observed difference in passive vessel dilatation following exposure to HS38, the intraluminal pressure‐dependent myogenic responses of HS38‐treated and DMSO vehicle control vessels were compared in the presence of 0 Ca^2+^ buffer (Figure 2c, i). HS38‐treated PCA vessels displayed distinct passive diameter responses to increased intraluminal pressure (*P* = 0.0001), showing significantly lower maximal diameters at pressures at or above 60 mmHg (Figure 2c, ii).

**FIGURE 3:**
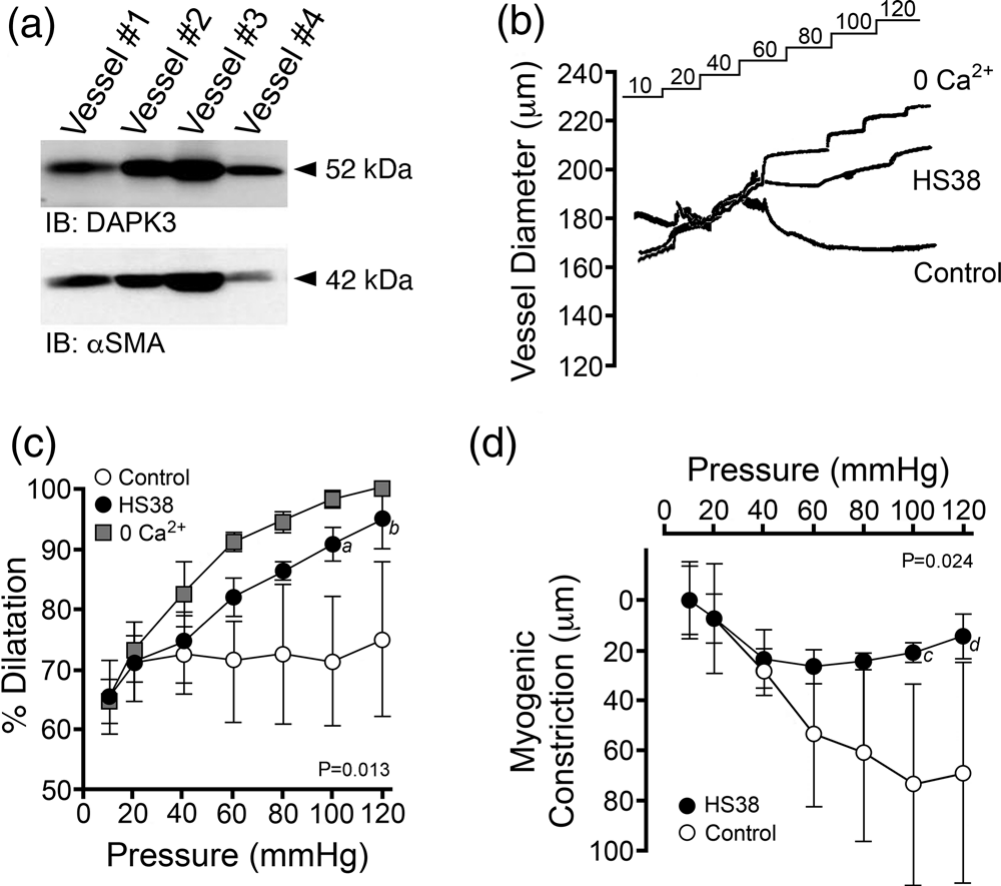
Myogenic responses of human cerebral arteries are attenuated with HS38 treatment. (a) Pial arteries (∼300–500 μm in diameter) were collected from four unique human brain tissue samples and immunoblotted for DAPK3 and smooth muscle actin (αSMA) as a loading control. (b) Representative recordings demonstrate the response of human pial arteries, outer vessel diameters, subjected to sequential 10–120 mmHg pressure steps in normal Krebs buffer (Control), with DAPK3 inhibitor (HS38, 10 μM), and in calcium‐free Krebs buffer (0 Ca^2+^). (c, d) Cumulative data provided to show the vessel constriction relative to the maximum passive diameter observed in 0 Ca^2+^ solution (c) and the magnitude of active myogenic constriction (d). HS38 treatment had a significant impact on percentage dilatation and active myogenic constriction at pressures above 100 mmHg; *P* = 0.013 and *P* = 0.024, respectively, by two‐way ANOVA with Šidák's multiple comparisons test (*a*, *P* = 0.007; *b*, *P* = 0.006; *c*, *P* = 0.013; *d*, *P* = 0.010. Data are presented as means with SD for vessels obtained from three different patients.

To determine if DAPK3 inhibition with HS38 has the potential to translate to the human vasculature, we assessed DAPK3 protein abundance as well as the myogenic response in a set of human cerebral arteries obtained from surgical samples of four different patients. Importantly, immunoreactivity for DAPK3 was detected in all samples examined (Figure 3a), suggesting HS38 may impact vascular contractility in these vessels. Myogenic responses were recorded in vessels isolated from three of the four tissue biopsies (Figure 3b). Basal myogenic reactivity varied somewhat for the three vessels; however, myogenic tone was developed at intraluminal pressures above 60 mmHg. This heterogeneity was expected as vessels were obtained from male and female patients of different ages with varied indications for surgery (e.g., epilepsy focal resection or tumour resection). The analysis of cumulative data showed the maximal myogenic constriction of human pial vessels to be 71.1 (13.5)% of the passive vessel diameter (Figure 3c). Myogenic tone was significantly inhibited with application of HS38 (*P* = 0.013), reducing maximal constriction to 94.9(6.7)% of maximal vessel diameter and active myogenic responses by approximately 50 μm at 100–120 mmHg (Figure 3d).

**FIGURE 4:**
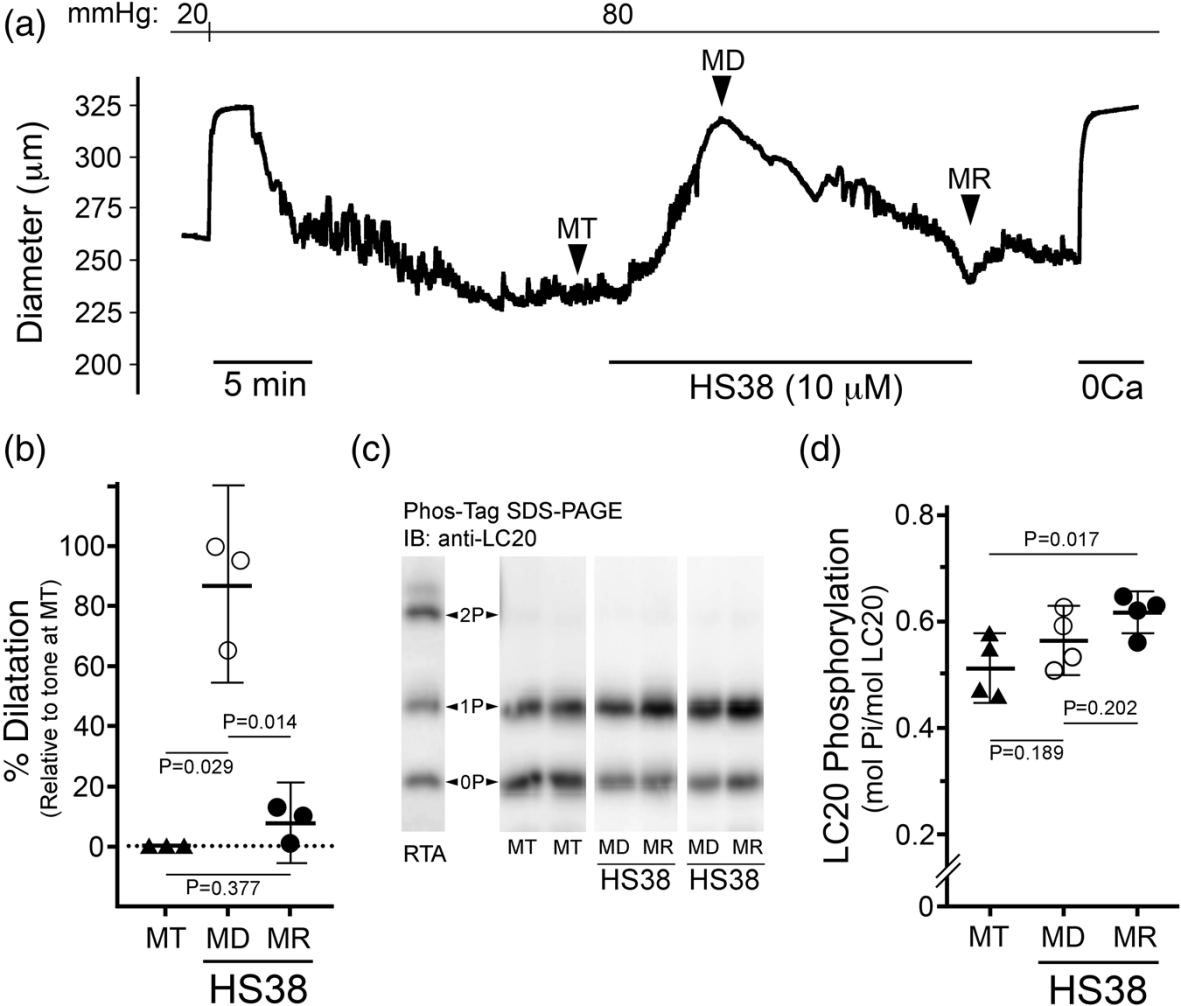
HS38 treatment of posterior cerebral arteries displaying active myogenic tone provides transient vasodilatation. (a) Representative vessel responses (outer diameter) to prolonged exposure with HS38. Vessels displayed active myogenic tone (MT) when pressurized to 80 mmHg. HS38 administration resulted in vessel dilatation (MD) and then spontaneous recovery of myogenic reactivity (MR). (b) Cumulative data show vessel dilatation in response to HS38; the percentage dilatation at MD and MR were calculated relative to the myogenic response observed at MT. Vessel dilatation at MD was significantly different from MT (*P* = 0.029) and MR (*P* = 0.014) while vessel dilatation at MR was not different from MT (*P* = 0.377); one‐way ANOVA and Tukey's multiple comparisons test. (c) Pressurized PCA vessels were flash‐frozen at the points denoted by the MT, MD and MR labels, and LC20 phosphorylation was determined by Phos‐tag SDS‐PAGE. A sample of rat tail artery (RTA) treated with microcystin was used to identify the phosphorylated LC20 (0P, unphosphorylated; 1P, monophosphorylated; 2P, dephosphorylated). (d) Cumulative data for LC20 phosphorylation stoichiometry are provided. Results were analysed by one‐way ANOVA and Tukey's multiple comparison test. LC20 phosphorylation at MD was not significantly different from MT (*P* = 0.189) or MR (*P* = 0.202); however, LC20 phosphorylation at MR was significantly different from MT (*P* = 0.017). Data are presented as means with SD; PCA vessels were obtained from *n* = 3–4 different male animals.

**FIGURE 5:**
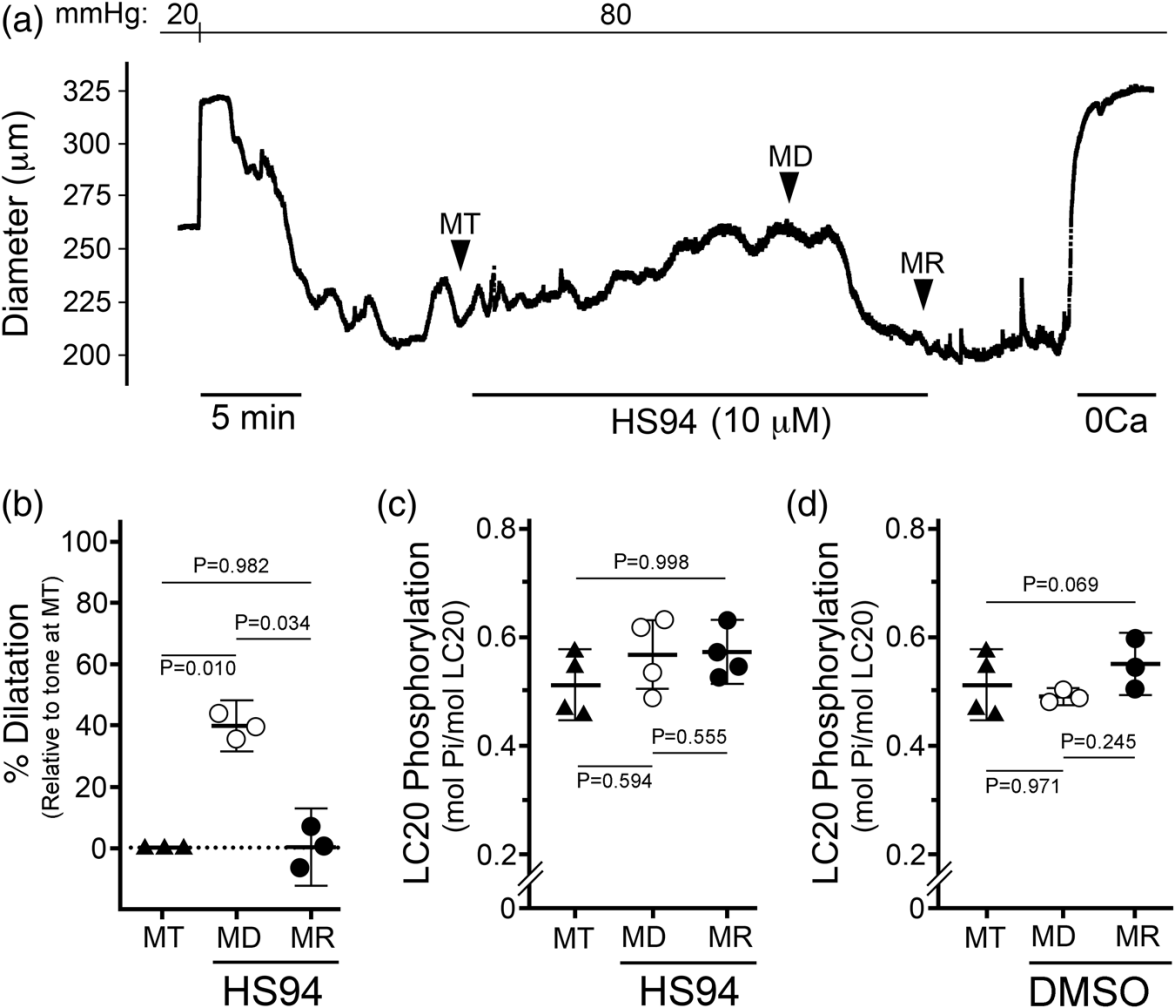
HS94 treatment of posterior cerebral arteries elicits transient vasodilatation. (a) Representative vessel responses (outer diameter) to prolonged exposure with HS94. Vessels displayed active myogenic tone (MT) when pressurized to 80 mmHg. HS94 administration resulted in vessel dilatation (MD) and then spontaneous recovery of myogenic reactivity (MR). (b) Cumulative data show vessel dilatation in response to HS94. Vessel dilatation at MD was significantly different from MT (*P* = 0.010) and MR (*P* = 0.034) while vessel dilatation after recovery at MR was not different from baseline tone at MT (*P* = 0.982); one‐way ANOVA and Tukey's multiple comparisons test. (c, d) Cumulative data for LC20 phosphorylation stoichiometry are provided for pressurized PCA vessels treated with HS94 or vehicle (DMSO), respectively. Vessels were flash‐frozen at the points denoted in (a), and LC20 phosphorylation stoichiometry was determined by Phos‐tag SDS‐PAGE as described in Figure 4. Results were analysed by one‐way ANOVA and Tukey's multiple comparison test. No significant differences in LC20 phosphorylation were identified: HS94 treatment: MD vs. MT (*P* = 0.594), MD vs. MR (*P* = 0.555), MT vs. MR (*P* = 0.998); DMSO treatment: MD vs. MT (*P* = 0.971), MD vs. MR (*P* = 0.245), MT vs. MR (*P* = 0.069). Data are presented as means with SD; PCA vessels were obtained from *n* = 3–4 different male animals.

In a subsequent set of experiments, endothelium‐denuded rat PCA vessels were maintained at an intraluminal pressure of 80 mmHg and provided continual HS38 exposure (Figure 4a). In this case (Figure 4b), the pharmacological action of HS38 was characterized by a biphasic response: first, a rapid inhibition of myogenic tone and nearly complete dilatation (85.0 (18.0)%; *P* = 0.029) of the pressurized vessel within 5 min; and second, a slower recovery of myogenic tone and restoration of the original vessel diameter over the next 20 min (8.2 (6.4)%; *P* = 0.014). As noted previously, some narrowing of vessels was also observed following HS38 when measuring the passive diameter in 0 Ca^2+^ solution at the experiment conclusion. As shown in Figure 4c, the dilatation induced by HS38 treatment was not associated with any decline in the level of LC20 phosphorylation (*P* = 0.189). However, a significant increase in LC20 phosphorylation was identified during the spontaneous recovery of myogenic tone and vessel constriction (*P* = 0.013). Additional myography was conducted with a second generation DAPK inhibitor compound (i.e., HS94, *K*_i_ 0.13 μM vs. HS38, *K*_i_ 0.26 μM; Carlson et al., 2018). Continuous exposure of pressurized PCA vessels to HS94 yielded a similar phenotype as HS38 (Figure 5a); a biphasic response was observed with vessel dilatation and then recovery of myogenic tone over time (Figure 5b). However, the kinetics of dilatation and recovery were qualitatively distinct from those observed with HS38, with a slower rate of vessel dilatation and a more rapid recovery of vessel constriction. As quantified in Figure 5c, LC20 phosphorylation was not significantly impacted with HS94 administration, either during the dilatation induced by HS94 administration (*P* = 0.594) or with the spontaneous recovery of myogenic tone (*P* = 0.550). No significant alterations in LC20 phosphorylation were observed for pressurized vessels sampled at similar time points with continual exposure to vehicle/DMSO (Figure 5d; *P* = 0.971 and *P* = 0.069). Taken together, these results suggest that distinct signalling pathways control vessel responses during the long‐term exposure to small molecule inhibitors of DAPK3 (i.e., HS38 and HS94).

**FIGURE 6:**
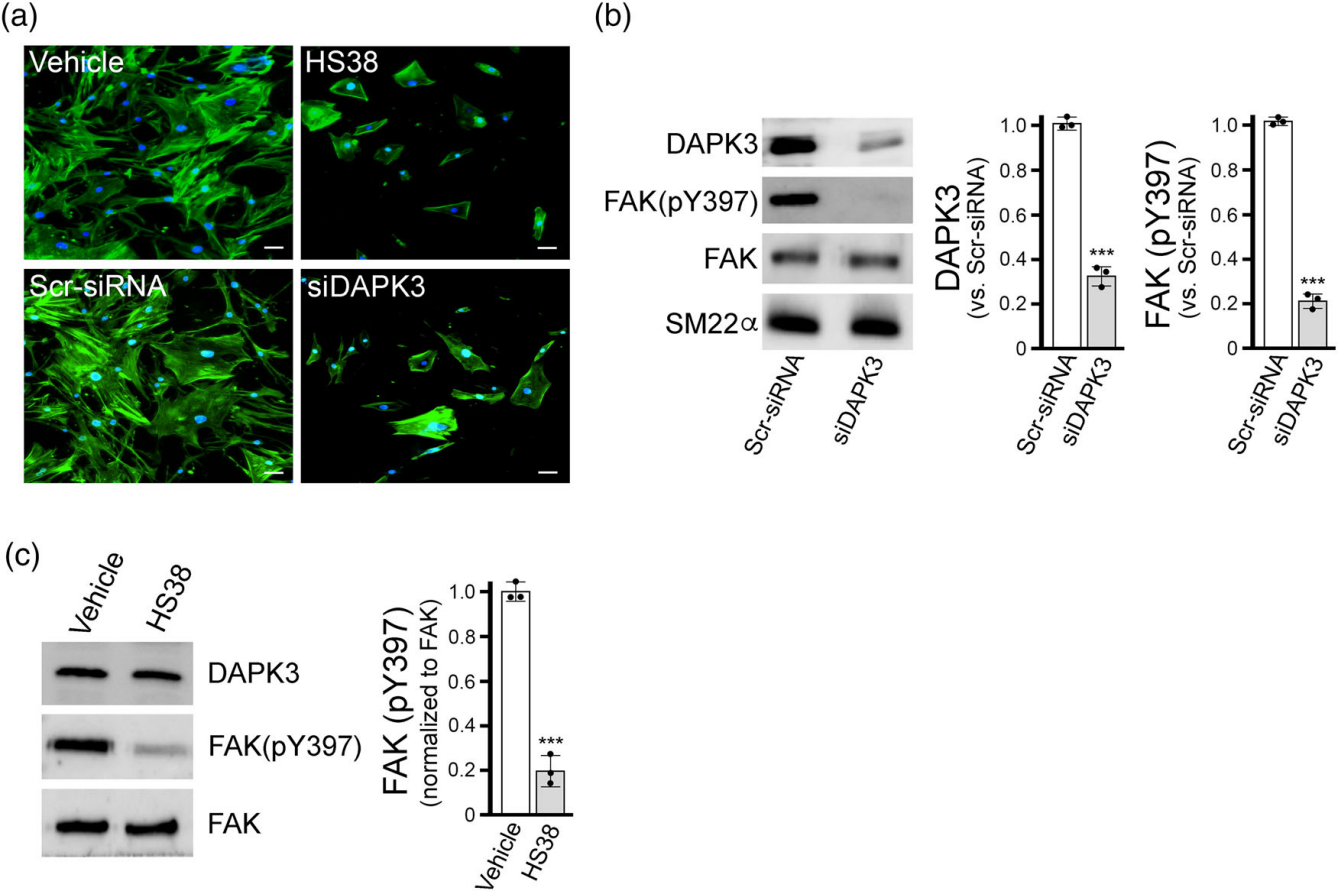
Effect of DAPK3 silencing on cytoskeletal architecture and FAK phosphorylation of vascular smooth muscle cells. (a) CASMCs were fixed and stained with AlexaFluor488‐phalloidin to examine F‐actin cytoskeletal organization following 16 h administration of small molecule inhibitor (HS38, 50 μM or DMSO vehicle control) or siRNA (siDAPK3 or scrambled (Scr)‐siRNA). Representative images from three independent experiments are shown with fluorescence signals for phalloidin‐F‐actin (green) and DAPI nuclear stain (blue). (b, c) Western blotting and densitometric quantification of total cell lysates was also used to examine FAK phosphorylation: DAPK3 and pY397‐FAK levels following treatment with siDAPK3 (b) and pY397‐FAK levels after HS38 administration (c). Data are means with SD from *n* = 3 different experiments using separate passages of cells. Significantly different from corresponding control, Student's *t*‐test, ***P < 0.0001.

Literature supports a role for DAPK3 in processes related to cytoskeletal organization (Chen & MacDonald, 2022), including actions on focal adhesion kinase (FAK) and adherens junctions (Nehru et et al., al., 2013; Zeng et al., 2021). Considering these reports, we further examined the impact of DAPK3 silencing on the cytoskeleton of human vascular SMCs. Marked changes in cell morphology as well as F‐actin stress fibre architecture were observed upon application of HS38 to CASMCs (Figure 6a). Similar observations were made upon DAPK3 knockdown using an siRNA approach. In this case, siDAPK3 treatments were found to decrease DAPK3 protein levels in CASMCs to 32 (4.7)% (*P* < 0.0001) of that found with control Scr‐siRNA treatments (Figure 6b). Stress fibres and cortical actin are regulated by FAK‐regulated processes (Mitra et al., 2005), so we examined the activating pY397 autophosphorylation site in the FERM domain that serves as a binding site for Src family kinases (Lietha et al., 2007). As presented in Figure 6b, the siRNA‐knockdown of DAPK3 in CASMCs was associated with a significant decrease in pY397‐FAK phosphorylation (*P* < 0.0001). Likewise, treatment of CASMCs with HS38 also significantly reduced the phosphorylation of pY397‐FAK (Figure 6c; *P* < 0.0001).

## 4 DISCUSSION

This is the first study to identify a role for DAPK3 in the myogenic response of the cerebral vasculature. We utilized the HS38 compound, a selective inhibitor that does not target other VSM contraction‐related kinases (Al‐Ghabkari et al., 2016; Carlson et al., 2013, 2018), to demonstrate the contribution of DAPK3 to the myogenic reactivity of PCA vessels. The HS38 inhibitor appears to impact myogenic reactivity through DAPK3‐dependent alterations to the cytoskeletal and focal adhesion dynamics of vascular SMCs, rather than via direct kinase actions on Ca^2+^ sensitization pathways (i.e., LC20 phosphorylation). Additional data support the translational importance of DAPK3 to the human cerebral vasculature, with robust expression of the protein kinase and significant HS38‐dependent attenuation of myogenic reactivity observed for pial vessels. Finally, intriguing pharmacokinetics were observed for the HS38 inhibitor that suggests intrinsic compensation by additional signalling pathways in response to the attenuation of DAPK3 activity during the maintenance of myogenic tone in the PCA.

The vascular myogenic response is a complex and highly regulated process that is the focus of much investigation (K. S. Hong et al., 2020; Jackson, 2021). Myogenic constrictions, which can occur in isolated endothelium‐denuded vessels in response to increasing luminal pressure, have a Ca^2+^ sensitive component, a Ca^2+^ sensitization component and an actin cytoskeletal reorganization component (Cole & Welsh, 2011; El‐Yazbi & Abd‐Elrahman, 2017; Osol et al., 2002; Walsh & Cole, 2013). Considerable evidence links DAPK3 to mechanisms of Ca^2+^ sensitization in smooth muscle contraction (Haystead, 2005; Ihara & MacDonald, 2007), namely, through the Ca^2+^‐independent phosphorylation of myosin regulatory light chain (LC20) or by the inhibition of myosin phosphatase (i.e., either by direct phosphorylation of the myosin phosphatase targeting subunit MYPT1 or by the phosphorylation of the myosin phosphatase inhibitor CPI‐17). Intriguingly, the dilatory responses of PCAs to HS38 occurred in the absence of corresponding changes to LC20 phosphorylation. The lack of change in LC20 phosphorylation with HS38 excludes contractile mechanisms involving a change in Ca^2+^ influx and cytosolic free [Ca^2+^] due to altered ion channel activity (e.g., decreased Ca^2+^ channel opening or increased K^+^ channel opening and hyperpolarization leading to indirect decline in cytosolic [Ca^2+^] and contraction); indeed, HS38 was previously identified to have no effect on the depolarization‐induced Ca^2+^ transients of VSM (MacDonald et al., 2016). It is also unlikely that the effects of HS38 on the dilatory responses of PCAs result from a change in myosin phosphatase activity through the inhibitory actions of rho‐associated protein kinase (ROCK) or CPI‐17, as both would result in a decrease in LC20 phosphorylation. This, as well as the observation that HS38 exposure elicits narrowing of PCA diameters under 0 Ca^2+^ conditions and high intraluminal pressures, suggests that DAPK3 does not regulate Ca^2+^ sensitization pathways during the myogenic response of vessels.

Mechanisms that provide Ca^2+^ sensitization of LC20 phosphorylation and force development may not be the only means for DAPK3 contributions to the myogenic character of PCA vessels. There is acknowledgement of a critical role of actin cytoskeleton remodelling in the development of myogenic tone (Cole & Welsh, 2011; Colinas et al., 2015; K. Hong et al., 2016; Ribeiro‐Silva et al., 2021; Walsh & Cole, 2013). Moreover, focal adhesion signalling via increased FAK Thr397 phosphorylation was required for the pressure‐dependent myogenic responses of cerebral arteries (Colinas et al., 2015). In alignment with these findings, DAPK3 is known to contribute to actin polymerization and focal adhesion dynamics with impact on the motility of vascular SMCs (Komatsu & Ikebe, 2004). Crosstalk between DAPK3 and FAK signalling in the regulation of focal adhesion dynamics was also observed for BJ/SV40 fibroblasts (Nehru et al., 2013). In these studies, over‐expression of a kinase‐dead DAPK3‐D161A mutant resulted in marked decrease in pY397‐FAK, but not pY576‐FAK phosphorylation. Our results also support of a role for DAPK3 in the regulation of FAK phosphorylation and actin cytoskeleton dynamics. In our studies, both the application of HS38 and the siRNA‐mediated knockdown of DAPK3 yielded similar attenuation of pY397‐FAK phosphorylation. There are some limitations to the comparison since the findings of Nehru et al. are reliant on over‐expression of DAPK3 variants in BJ/SV40 fibroblasts whereas our findings are dependent upon pharmacological and siRNA‐dependent silencing of DAPK3 in a vascular SMC.

RhoA/ROCK signalling is also involved in the actin polymerization that accompanies myogenic constrictions of cremaster arterioles (Moreno‐Dominguez et al., 2013), which may indicate a role for DAPK3 downstream of ROCK. In non‐smooth muscle cells, ROCK can phosphorylate DAPK3 at Thr265 and Thr299, leading to its activation and cytosolic localization (Hagerty et al., 2007). Considering the potent inhibitory effect of ROCK‐specific inhibitors on the myogenic response (Johnson et al., 2009; Moreno‐Dominguez et al., 2013), it is possible that DAPK3 may be involved downstream of ROCK to influence the remodelling of the actin cytoskeleton during myogenic constriction, and this relationship will require further investigation. In addition, DAPK3 was positioned downstream of G protein‐coupled receptors (GPCRs) that signal through Gq/11 and/or G12/13, and findings implied that the activation of RhoA/ROCK was required for subsequent DAPK3‐dependent events (MacDonald et al., 2016). These signalling linkages coincide with emerging evidence that identify the angiotensin II receptor (AT_1_R) and Gq/11‐coupled signals to function as sensors of membrane stretch in VSM cells (K. Hong et al., 2016; Kauffenstein et al., 2012; Mederos y Schnitzler et al., 2008; Pires et al., 2017). With specific DAPK3 and ROCK inhibitors now available, the mechanoactivation of AT_1_R and subsequent signalling through ROCK and DAPK3 should be thoroughly evaluated in the myogenic response.

HS38 has excellent potency and specificity for DAPK3 and, unlike other DAPK3 inhibitor compounds, exhibits no off‐target actions on Rho‐associated protein kinase (Al‐Ghabkari et al., 2016). Although HS38 is highly specific for DAPK3 when compared to other contractile kinases in smooth muscle (Carlson et al., 2013), the compound does exhibit polypharmacology with equal efficacy against the closely related DAPK1 and the Provirus integrating site Moloney murine leukaemia virus (PIM3) kinase. Some studies have linked DAPK1 to cytoskeletal proteins and motility, cell polarization and migration, as well as mechanosensing and matrix adhesion of fibroblasts (Chen & MacDonald, 2022); however, there is no evidence for DAPK1 signalling involvement in smooth muscle contractile processes. Indeed, the cellular expression of DAPK3 appears uniquely restricted when compared with the other DAPK family members. Any role for PIM3 in vascular SMC contraction remains to be defined. The PIMs are considered oncogenic Ser/Thr protein kinases with an expansive scope of biological impact on cell growth, proliferation and survival, cell differentiation, apoptosis, and metabolism (Sawada et al., 2006). More recent studies demonstrate PIM involvement in cancer cell migration and metastatic invasion, and PIM3 was also shown to colocalize with FAK at the lamellipodia (Zhang et al., 2009). In vitro biochemical assessments identified MYPT1 and LC20 to be substrate targets of PIM3 (Carlson et al., 2018). Ultimately, it remains to be determined whether PIM3 also impacts upon the actin cytoskeleton of vascular SMCs.

A few study limitations should be considered: (1) the polypharmacology of HS38 necessitates additional chemical refinement of the pharmacophore to ensure the mitigation of off‐target effects; (2) the mechanism(s) whereby DAPK3 regulates the Y397 phosphorylation status of FAK remains to be defined, but it is unlikely this is a direct phosphorylation of FAK by DAPK3; (3) DAPK3‐mediated effects on the actin cytoskeleton and focal adhesions were demonstrated in cultured CASMCs, but these findings remain to be confirmed during the myogenic responses of isolated cerebral vessels; (4) as the dominant off‐target of HS38, PIM3's expression and potential role in myogenic reactivity requires additional interrogation; and (5), experimentation should be conducted to address any potential sexual dimorphism of DAPK3 in the myogenic responses of vessels isolated from males and females. Supporting Information

## AUTHOR CONTRIBUTIONS

All persons designated as authors qualify for authorship, and all those who qualify for authorship are listed. Sara R. Turner, Abdulhameed Al‐Ghabkari, Mona Chappellaz, and Cindy Sutherland completed the data analysis and prepared figures. David A. Carlson synthesized the HS38 and HS94 compounds. Timothy A. J. Haystead coordinated the production of inhibitor compounds and made intellectual contributions to the project. William C. Cole made intellectual contributions to the project, assisted in data interpretation and reviewed the manuscript. Justin A. MacDonald conceived and coordinated the study, assisted with experimental design, wrote the manuscript, provided trainee supervision, and made intellectual contributions to the project. All authors approved the final version of the manuscript and agree to be accountable for all aspects of the work in ensuring that questions related to the accuracy or integrity of any part of the work are appropriately investigated and resolved. All persons designated as authors qualify for authorship, and all those who qualify for authorship are listed.

## CONFLICT OF INTEREST

J.A.M. is cofounder and has an equity position in Arch Biopartners Inc. T.A.J.H. is founder and has an equity position in Eydis Bio Inc. All other authors declare no conflicts of interest.

## Supporting information

Statistical Summary Document

Dataset

## Data Availability

The data that support the findings of this study are available from the corresponding author upon reasonable request.
